# A method for measuring first glymphatic influx of a cerebrospinal fluid tracer in the human brain

**DOI:** 10.3389/fnins.2025.1703748

**Published:** 2025-12-19

**Authors:** Are Hugo Pripp, Geir Ringstad, Lars Magnus Valnes, Per Kristian Eide

**Affiliations:** 1 Oslo Centre of Biostatistics and Epidemiology, Oslo University Hospital, Oslo, Norway; 2 KG Jebsen Centre for Brain Fluid Research, University of Oslo, Oslo, Norway; 3 Faculty of Health Sciences, OsloMet—Oslo Metropolitan University, Oslo, Norway; 4 Department of Radiology, Oslo University Hospital-Rikshospitalet, Oslo, Norway; 5 Department of Geriatrics and Internal Medicine, Sorlandet Hospital, Arendal, Norway; 6 Institute of Clinical Medicine, Faculty of Medicine, University of Oslo, Oslo, Norway; 7 Department of Neurosurgery, Oslo University Hospital-Rikshospitalet, Oslo, Norway; 8 Department of Mathematics, University of Oslo, Oslo, Norway

**Keywords:** cerebral cortex, glymphatic system, magnetic resonance imaging, regression analysis, white matter

## Abstract

**Introduction:**

The glymphatic system is a brain-wide perivascular transport route for fluids and solutes in which cerebrospinal fluid (CSF) serves as a conduit for solute transport and clearance of brain waste. Intrathecal contrast-enhanced magnetic resonance imaging (MRI), where the intrathecal contrast agent serves as a CSF tracer, has been developed to measure glymphatic function in humans. The normalized MRI T1 signal is a semiquantitative measure of CSF flow and exchange with the brain. Objective: To estimate first-time tracer appearance within brain tissue after intrathecal tracer injection.

**Methods:**

This study implemented segmented regression analysis to estimate the first-time tracer appearance of an intrathecal tracer within brain tissues. An increase (breakpoint) in the normalized MRI T1 signal was defined to represent first glymphatic influx of the tracer. The study included 30 reference (REF) subjects with no identified CSF disturbance and 15 patients with a diagnosis of idiopathic intracranial hypertension (IIH). We developed and evaluated the method in REF subjects and further compared it between the two study groups.

**Results:**

The time to initial glymphatic tracer enrichment in the REF cohort was approximately 1 h in the frontal, temporal, parietal, and occipital cerebral cortex and ranged from two to 4 h in the corresponding white matter regions. In subcortical limbic structures and basal ganglia structures, it was 0.6 and 2.2 h, respectively. Compared with REF subjects, IIH patients presented a non-significant mean difference in the first appearance of ±0.5 h in the cerebral cortex and white matter regions, with somewhat longer estimated delays in the parietal and insular white matter regions. The results are presented as time series plots and estimates with 95% confidence intervals. Additionally, we provide supplementary R code, which can be adapted for use in future studies, and outline a basic assessment of true versus estimated breakpoints using simulated data.

**Conclusion:**

Segmented regression was found feasible to quantify the time to first glymphatic enrichment, i.e., increase in the normalized MRI T1 signal. Moreover, the method seems reasonable to differentiate first glymphatic influx between the cohorts.

## 1 Introduction

The glymphatic system is a brain-wide perivascular transport route for fluids and solutes, including nutrients, drugs, and metabolic waste, in which cerebrospinal fluid (CSF) exchange with the brain is crucial for this function (Rasmussen et al., 2022). Large CSF compartments, such as the subarachnoid space (SAS) and cerebral ventricles, are in direct communication with smaller compartments, such as the perivascular spaces, along the richly vascularized brain. This enables CSF from the SAS to pass deep into the brain along periarterial spaces. According to the glymphatic concept (Nedergaard and Goldman, 2020; Rasmussen et al., 2022; Kelley and Thomas, 2023), the CSF mixes with the interstitial fluid, passes through the extracellular space, and thought to exit the brain via perivenous spaces. Several aspects of the glymphatic concept are still under debate (Hladky and Barrand, 2022) including recent debates about brain clearance of metabolites and toxins during sleep (Miao et al., 2024). However, several lines of experimental evidence, including live imaging using two-photon microscopy and magnetic resonance imaging (MRI), strongly support the glymphatic concept along arteries (Iliff et al., 2013; Yang et al., 2013; Kipnis et al., 2025).

Our research group translated the experimental findings from animals to humans and showed that an intrathecally administered MRI contrast agent utilized as a CSF tracer produced brain-wide enrichment of the extravascular compartment, mainly in the cortex (Eide and Ringstad, 2015; Ringstad et al., 2018). T1-weighted MRI revealed increased tracer enrichment and delayed clearance from a wide range of brain regions in idiopathic intracranial hypertension (IIH) patients (Eide et al., 2021b), impaired clearance in patients with a dementia subtype (idiopathic normal pressure hydrocephalus, iNPH) (Ringstad et al., 2017; Eide et al., 2021b) and impaired CSF clearance following one night of total sleep deprivation (Eide et al., 2021c). More recent evidence has shown that glymphatic influx (i.e., influx of the contrast agent serving as CSF tracer) is preceded by the CSF tracer following the pathways of a compartmentalized SAS, namely, the perivascular subarachnoid spaces (PVSAS), which facilitate antegrade transport along the arteries toward the brain and glymphatic perivascular spaces (Eide and Ringstad, 2024). Furthermore, early glymphatic influx was positively correlated with enrichment within the PVSAS and delayed in both compartments of iNPH patients.

In this context, there is a need to develop improved methodologies to measure early glymphatic influx utilizing intrathecal contrast-enhanced MRI. In our previous studies, we mostly assessed glymphatic function during the clearance phase, 24 to 48 h after tracer injection (Eide et al., 2021a), but we have not addressed how to assess variability in first glymphatic influx within a few hours. Specifically, we aimed to develop a statistically based method to measure the time to the first appearance of a glymphatic tracer across brain segments.

Here, we utilize a statistical model known as segmented regression, which may be used to analyze chronologically ordered series of repetitive observations over time that are “interrupted” at a specific time point by an event (Schober and Vetter, 2021). Segmented regression essentially models the trend of the outcome over time and, in its simplest form, fits two distinct lines to the data: one before the “interruption” and one after (Muggeo, 2003; Muggeo, 2008). In this context, the “interruption” causing a breakpoint in the lines is assumed to represent the change in the MRI signal that marks the first detection of the glymphatic tracer. The statistically estimated time at which this change or breakpoint in the MRI signal occurs provides an estimate of the time from injection to the first detection of glymphatic tracers in brain regions. To the best of our knowledge, segmented regression has not yet been reported as a statistical technique for assessing glymphatic influx and there is no validated “gold standard” method to quantify time to first glymphatic influx. In this study, which included patients who underwent intrathecal contrast-enhanced MRI, we developed and evaluated a statistical estimate of the period between injection and the first detection of the tracer in brain regions using segmented regression models in reference (REF) subjects. Additionally, we compared the time to the first appearance of the tracer in the brain between the two diagnostic groups in the study cohort: REF subjects and IIH patients.

## 2 Materials and methods

### 2.1 Study cohort

This study included consecutive patients with symptoms rendering for a broad clinical examination including intrathecal contrast-enhanced MRI as part of their neurosurgical work-up within the Department of Neurosurgery at the Oslo University Hospital, Norway. Intrathecal contrast-enhanced MRI was performed in individuals who underwent a broad work-up for CSF tentative disturbance. The present study cohort included individuals in whom the diagnostic algorithms disclosed no CSF or neurological disease, denoted REF subjects, and IIH patients who were offered this diagnostic work-up and in whom conservative treatment had failed (denoted IIH cohort). The following criteria for IIH were used: increased lumbar opening pressure (>25 mmH2O), papilledema, normal CSF composition, normal MRI with exclusion of venous thrombosis and normal neurological examination (except for fourth cranial nerve affection) (Mollan et al., 2018). We have previously reported on these patients (Ringstad et al., 2018; Eide et al., 2021a; Eide et al., 2021b) but did not use the present algorithm, and none of the results of the present analysis have been reported.

### 2.2 Assessment of tracer enrichment in CSF

The MRI contrast agent gadobutrol was used as a CSF tracer. To assess the influx of the CSF tracer gadobutrol in the human brain, an interventional neuroradiologist administered gadobutrol intrathecally at the lumbar level at a dose of 0.5 mmol (0.5 mL of 1.0 mmoL/mL gadobutrol; Gadovist, Bayer Pharma AG, Berlin, Germany). Standardized T1-weighted MR scanning was performed before and at multiple time points after intrathecal gadobutrol injection, such as 0, 2, 4, 6, 24, and 48 h, see Supplementary Figure 1. Gadobutrol enhances the T1 relaxation of water and thereby enhances the T1 signal intensity at the image grayscale. It provides a semiquantitative measure of the tracer level. We used FreeSurfer software (version 6.0)^1^ for image postprocessing to determine the percentage change in the normalized T1 signal units that indicate CSF tracer influx.

### 2.3 MRI protocol and image analysis

MRI was performed in a 3 Tesla Philips Ingenia MRI scanner (Philips Medical systems, Best, The Netherlands) with identical imaging protocol settings to acquire sagittal 3D T1-weighted volume scans. The imaging parameters were set as follows: repetition and echo time were typically 5.1 ms and 2.3 ms, respectively, the flip angle was 8 degrees, and the field of view was 256 × 256 cm with a matrix resolution of 256 × 256 pixels (reconstructed 512 × 512). We sampled 184 overlapping slices with a thickness of 1 mm, which were automatically reconstructed into 368 slices with a thickness of 0.5 mm.

FreeSurfer software (Fischl, 2012) was used for segmentation, parcellation of the pre-contrast MR images and registration/alignment of the longitudinal data, which allowed for analysis of the increase in T1 intensity caused by the CSF tracer (Ringstad et al., 2018). The process involves removing non-brain tissue and fluid spaces at the brain surface were removed via a hybrid watershed/surface deformation procedure (Segonne et al., 2004). Followed by automated Talairach transformation, and segmentation of the subcortical white matter and deep gray matter volumetric structures (including hippocampus, amygdala, caudate, putamen, and ventricles; Fischl et al., 2002), and parcellation based on brain atlas (Fischl et al., 2004). The alignment process for each subject involves creating a median template of MR images registered to the pre-contrast MR image (Reuter et al., 2012), and registering all MR images for that subject to the template MR image (Reuter et al., 2010). The segmentation and registration was manually checked by LMV for any significant errors. The MR images were adjusted for changes in the grayscale between MRI scans by dividing the T1 signal unit for each time point by the T1 signal unit of a reference region of interest (ROI) for the respective time point.

The reference ROI was placed within the posterior part of the orbit, as previously described (Eide et al., 2021c). The normalized T1 signal unit thus corrects for baseline changes in the image grayscale due to automatic image scaling.

### 2.4 Statistical analysis

The statistical analysis was performed using R (version 4.4.2, R Project for Statistical Computing), RStudio (version 2024.12.1, Posit Software, PBC), Stata/MP statistical software (version 18.0, StataCorp LLC) and the segmented package (Muggeo, 2008). Continuous data are reported as the mean with the standard deviation (SD), whereas categorical data are presented as the number of observations and are statistically assessed using Student’s two-sample t test or Pearson’s chi-square test, respectively.

MRI scans were conducted once before intrathecal tracer injection for all patients, but the number and time of subsequent scans after tracer injection varied among participants because they were obtained from inpatient clinical examination. Therefore, the individual first detected increase in tracer signal would be influenced by the study design and time of the MRI scans. For the segmented regression analysis, we used MRI scans from the image analysis database covering the time range before and the first day after injection (i.e., until approximately 7 hours after injection). While measurements taken approximately 24 h postinjection were visually inspected, they were not included in the statistical analysis.

Segmented regression models are a type of regression analysis in which the relationship between the response variable and one or more explanatory variables is piecewise linear. This means that it consists of segments joined at points called breakpoints. We assumed an initial horizontal line until the first breakpoint and a change in slope due to the first tracer signal. The time of the first breakpoint with a 95% confidence interval (CI) was interpreted as a measure of first-time glymphatic tracer enrichment after injection. The model iteratively estimated the optimal breakpoint, with its 95% CI, within the series of measurements.

These analyses were conducted by combining predefined FreeSurfer segments into the frontal, temporal, parietal and occipital cerebral cortex and respective white matter regions and other selected regions as initially outlined by Ringstad et al. (2018). We assessed the study cohort of REF subjects and IIH patients separately, reporting one breakpoint per brain region. The mean differences in first-time tracer appearance between the REF and IIH groups with 95% CIs and *p* values were determined using a z test based on the standard errors from the estimated breakpoints, and *p*-values were assessed for multiplicity using Holm’s method to control the family-wise error rate. Time series plots of the normalized T1 MR signal with the fitted model were visually inspected. The CI width expresses the precision of the estimates. Additionally, we explored an expanded statistical model to assess differences between the two diagnostic groups by modeling a common initial horizontal line but with separate breakpoints for each study group.

Examples of R code using simulated data are included in the Supplementary material for both one breakpoint of a study cohort and separate breakpoints for two study groups. We also present a basic assessment of true versus estimated breakpoints in different scenarios using simulated data.

## 3 Results

### 3.1 Participant characteristics

The study included 45 participants, with 30 REF subjects and 15 IIH patients. As presented in Table 1, the IIH group was similar in terms of age and proportion of men and had a somewhat higher body mass index (BMI) than the REF group.

**Table 1:** Demographic overview of the participants.

	Patient category			Significance
	Overall	REF	IIH	
Number of subjects	45	30	15	
Sex (male/female)	8/37	6/24	2/13	0.669
Age (years)	36.2 ± 11.3	36.9 ± 11.0	34.9 ± 12.1	0.582
BMI (kg/m^2^)	28.3 ± 5.4	26.8 ± 5.2	31.1 ± 4.7	0.012

**Figure 1:**
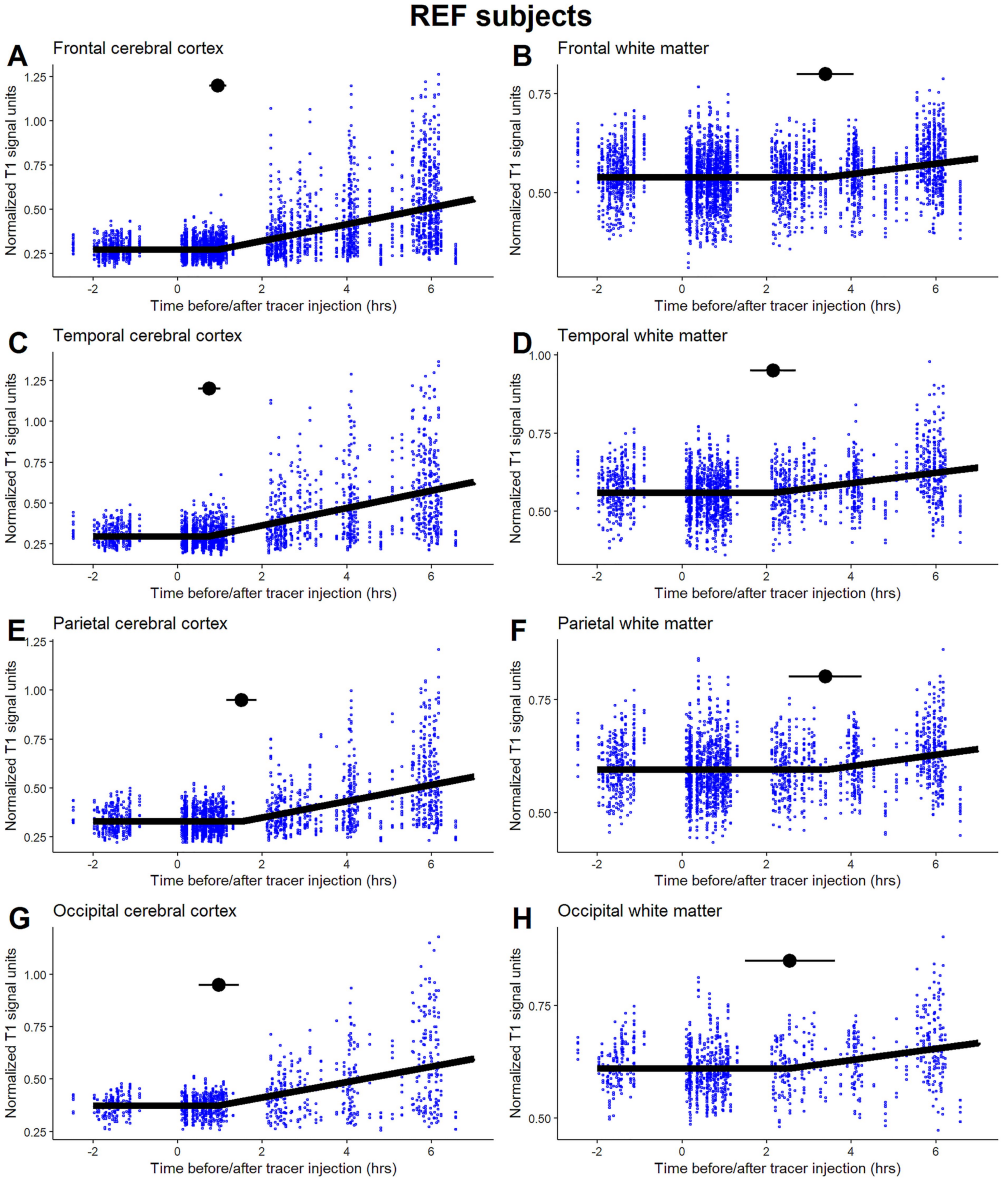
Time-series plots with the fitted segmented regression model for the reference (REF) subjects. The time to first detection of the tracer expressed by the breakpoint is indicated by horizontal point intervals in **A** and **B**) the frontal cerebral cortex and white matter, **C** and **D**) the temporal cerebral cortex and white matter, **E** and **F**) the parietal cerebral cortex and white matter, and **G** and **H**) the occipital cerebral cortex and white matter. Individual data points from the participants are shown for transparency and to express the data variation.

**Figure 2:**
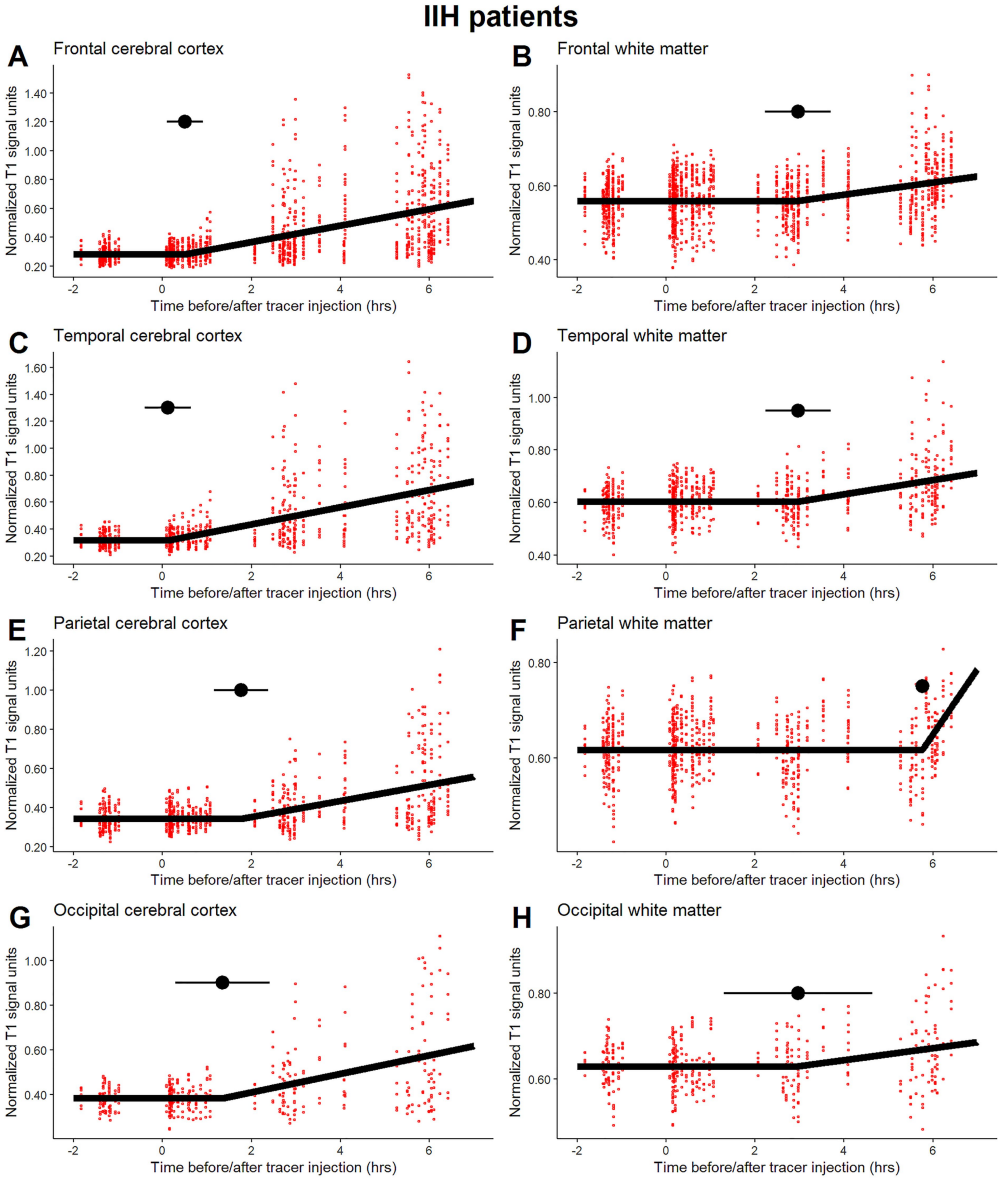
Time-series plots with the fitted segmented regression model for idiopathic intracranial hypertension (IIH) patients. The time to first detection of the tracer expressed by the breakpoint is indicated by horizontal point intervals in **A** and **B**) the frontal cerebral cortex and white matter, **C** and **D**) the temporal cerebral cortex and white matter, **E** and **F**) the parietal cerebral cortex and white matter, and **G** and **H**) the occipital cerebral cortex and white matter. Individual data points from the participants are shown for transparency and to express the data variation.

**Figure 3:**
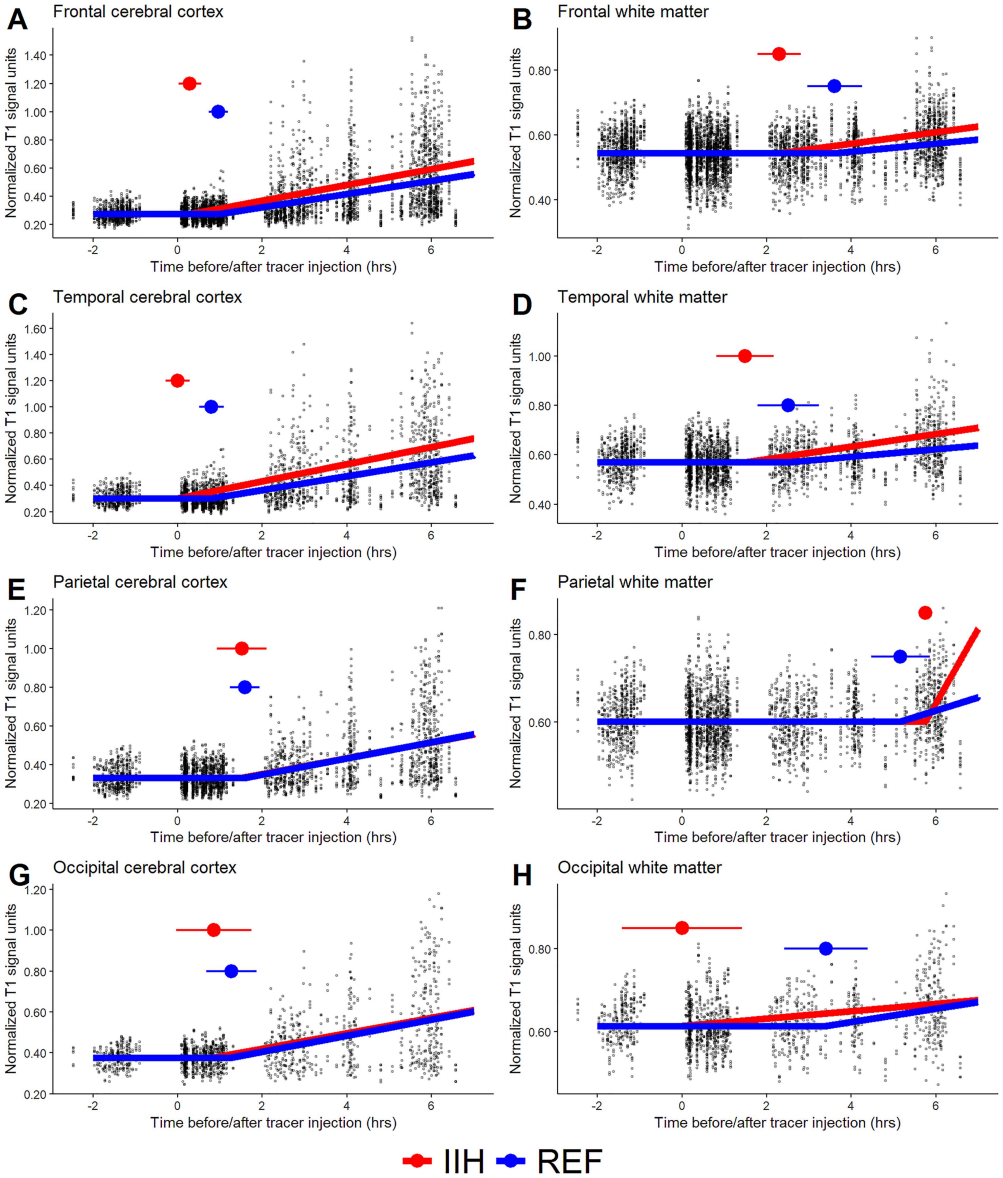
Time-series plots with the fitted segmented regression model from the expanded statistical approach. It included the two diagnostic groups in the same statistical model with a common horizontal line before tracer injection and until the respective breakpoints representing the estimated first-time appearance of the tracer in each group. Patient categories: *REF,* reference cohort; *IIH*, idiopathic intracranial hypertension.

The data are presented as the number of observations or the mean ± SD. Differences between groups were determined by Student’s two-sample t test for continuous variables and by Pearson’s chi-square test for categorical variables. The number of MRI scans before and after injection was 32 and 177 in the REF group, and 15 and 55 in the IIH group. The mean (SD) time of measurement, as reported in Figures 1–3, was similarly distributed with 1.6 (2.3) and 1.6 (2.5) in the REF and IIH participants, respectively. A time point before injection has a negative value in the estimation of the mean and standard deviation. Patient categories: *REF* reference cohort, *IIH* idiopathic intracranial hypertension.

### 3.2 Estimating first-time tracer detection with segmented regression

Our statistical approach for assessing first-time tracer detection (i.e., glymphatic influx) is illustrated by time series plots of major cerebral cortex and white matter regions from REF subjects with fitted segmented regression models in Figure 1. The estimated breakpoint with 95% CI produced is proposed as an estimate of the time to first tracer appearance (glymphatic influx), as indicated in each plot with a horizontal point interval. The individual data points from the participants are shown for transparency and to express the data variation.

A participant individual estimation of breakpoints in the segmented regression was explored using a random breakpoint modeling approach. Due to the large variation in the time span between MRI scans and the number of measurements for the participants, this approach was not applicable (results not shown). A reasonable number of data points, which are equally distributed during the follow-up period, improve the performance of the segmented regression method in assessing the first appearance of the tracer. MRI scans were conducted as part of a clinical examination, which explains the lack of data from approximately 7 h after injection until 24 h, as scans were typically performed the next morning. Using the measurements after 24 h in the statistical model was not feasible, but they were visually assessed during the initial phase of the statistical modeling to confirm the later appearance of the tracer. All participants showed tracer enrichment at 24 h, and none were excluded based on visual assessment.

### 3.3 First-time tracer appearance in REF and IIH participants

The time from injection to the first breakpoint with the corresponding 95% CI derived from linear segmented regression models was estimated for the main brain region from IIH patients (Figure 2). The estimated first-time tracer appearance is summarized in Table 2, including other selected regions and the mean difference between the REF subjects and IIH patients with 95% CIs and *p* values. Estimated *p*-values that were also statistically significant at the 5% level after Holm’s method to control for multiplicity are marked. The time before initial glymphatic tracer enrichment in the frontal, temporal, parietal and occipital cerebral cortex was approximately 1 h and ranged until approximately 5 h in the corresponding white matter regions. Other selected cerebral cortex and white matter regions showed similar results, with an approximately two-hour delay in white matter compared with the cerebral cortex (Table 2). The first appearance times of the subcortical limbic and basal ganglia structures in REF subjects were 0.6 and 2.6 h, respectively. This quantifies the delay in first tracer appearance between the cerebral cortex and white matter regions and differences in subcortical structures.

**Table 2:** First-time tracer appearance (hours) with 95% CI and mean difference between the study groups.

Anatomical region	REF	R^2^	IIH	Mean difference (IIH–REF)	R^2^
Major brain regions					
Frontal cerebral cortex	0.9 (0.7 to 1.2)	0.36	0.5 (0.1 to 0.9)	−0.5 (−0.9 to 0.0), *p* = 0.052	0.35
Frontal white matter	3.4 (2.7 to 4.0)	0.03	3.0 (2.2 to 3.7)	−0.4 (−1.4 to 0.6), *p* = 0.412	0.08
Temporal cerebral cortex	0.7 (0.5 to 1.0)	0.35	0.1 (−0.4 to 0.6)	−0.6 (−1.2 to 0.0), *p* = 0.035	0.37
Temporal white matter	2.1 (1.6 to 2.7)	0.09	3.0 (2.2 to 3.7)	0.8 (−0.1 to 1.7), *p* = 0.078	0.14
Parietal cerebral cortex	1.5 (1.1 to 1.9)	0.29	1.8 (1.2 to 2.4)	0.3 (−0.4 to 1.0), *p* = 0.472	0.26
Parietal white matter	3.4 (2.5 to 4.2)	0.03	5.8 (5.6 to 5.9)	2.4 (1.5 to 3.3), *p* < 0.001	0.06
Occipital cerebral cortex	1.0 (0.5 to 1.5)	0.28	1.4 (0.3 to 2.4)	0.4 (−0.8 to 1.5), *p* = 0.522	0.26
Occipital white matter	2.6 (1.5 to 3.6)	0.07	3.0 (1.3 to 4.6)	0.4 (−1.6 to 2.4), *p* = 0.679	0.06
Other selected regions					
Entorhinal cerebral cortex	0.3 (−0.1 to 0.7)	0.65	−0.1 (−0.8 to 0.6)	−0.4 (−1.2 to 0.3), *p* = 0.273	0.69
Entorhinal white matter	2.0 (1.1 to 2.8)	0.22	2.4 (1.7 to 3.2)	0.5 (−0.7 to 1.6), *p* = 0.424	0.45
Insular cerebral cortex	0.6 (0.3 to 0.9)	0.69	0.1 (−0.7 to 0.8)	−0.5 (−1.3 to 0.2), *p* = 0.175	0.67
Insular white matter	2.3 (0.7 to 3.9)	0.06	5.7 (5.5 to 6.0)	3.4 (1.8 to 5.0), p < 0.001	0.18
Cingulate cerebral cortex	0.7 (0.5 to 0.9)	0.53	0.3 (−0.2 to 0.8)	−0.5 (−1.0 to 0.1), *p* = 0.106	0.49
Cingulate white matter	2.2 (1.3 to 3.0)	0.06	3.0 (1.7 to 4.2)	0.8 (−0.7 to 2.3), *p* = 0.281	0.08
Basal ganglia	2.2 (0.8 to 3.5)	0.02	2.9 (0.8 to 4.9)	0.7 (−1.7 to 3.2), *p* = 0.567	0.03
Limbic structures	0.6 (0.3 to 0.8)	0.33	0.0 (−0.5 to 0.5)	−0.6 (−1.1 to −0.1), *p* = 0.027	0.40
Choroid plexus	0.2 (−0.7 to 1.1)	0.25	−1.1 (−3.3 to 1.1)	−1.2 (−3.6 to 1.1), *p* = 0.304	0.25

*Also significant at the 5% level after controlling for multiplicity using Holm’s method. R^2^: Coefficient of determination.

Figure 3 shows the expanded statistical approach, including two diagnostic groups in the same statistical model, with a common horizontal line before tracer injection and until the respective breakpoints representing the estimated first-time appearance of the tracer in each diagnostic group. This statistical approach could be affected by minor systematic differences in signals before tracer detection (i.e., the common horizontal line), which might explain the somewhat unexpected early tracer detection of occipital white matter in IIH patients (Figure 3H). Thus, separate statistical modeling for each diagnostic group might be better and is therefore reported in Table 2. The two cohorts showed a nonsignificant difference of ± 0.5 h in the first tracer appearance in most regions, except for a significant delay in IIH patients in parietal white matter and insular white matter of 2 to 3 h compared with REF subjects (Table 2). Overall, the models had better model fit, as given by the coefficient of determination (R^2^), in cerebral cortex compared to white matter regions.

The data are presented as first-time tracer appearance (hours) with 95% CIs estimated through segmented regression modeling (Figures 1,2) and the mean difference in first-time tracer appearance between the REF and IIH groups with 95% CIs and *p* values determined using a z test based on the standard errors from the estimated breakpoints. Patient categories: *REF* reference cohort, *IIH* idiopathic intracranial hypertension.

### 3.4 R codes with simulated data

As Supplementary material (Additional file 1), examples of R codes that estimate a breakpoint to represent the first appearance of a tracer in one study group and separate breakpoints for two study groups (as used in Figure 3) are shown. In addition, a basic time series plot with data points and fitted segmented regression models from the simulated data are added (Supplementary Figures 2, 3). Simulated data are used to demonstrate the method. Furthermore, Supplementary Figure 4 outlines scenarios, based on the model shown in Supplementary Figure 2, comparing true versus estimated breakpoints with an increasing number of data points and different time distributions. Analysis of the simulated data indicates that the estimated breakpoint from the segmented regression modeling closely corresponds to the statistically expected value. As expected, the relationship between true and estimated breakpoints improved with an increasing number of data points (Supplementary Figure 4). A few comments have been added to the R codes to facilitate implementation in other research projects.

## 4 Discussion

Segmented regression analysis seemed feasible to estimate the time to first appearance of the tracer, as a breakpoint occurs in the T1 signal unit following the intrathecal injection of a CSF tracer. The estimated time likely represents the time interval necessary for the CSF tracer to first enrich the various brain regions. As such, it can serve as a proxy for assessing the efficiency of glymphatic influx. At the one-day follow-up, tracer enhancement was observed in all regions (data not shown), which is consistent with previous reports from this cohort (Eide et al., 2021a; Eide et al., 2021b). In addition, the steepness of the slope after the breakpoint may serve as a proxy measure of glymphatic activity and brain fluid flow; however, this requires further investigation.

The study of the glymphatic system has important clinical implications for understanding the healthy brain as well as neurological diseases; evaluating statistical methods for analyzing data from studies of glymphatic function should be a part of this research.

Our findings highlight the slower glymphatic enrichment in deep brain regions. Previous studies revealed tracer enhancement in CSF spaces within 1 h before enhancement in the cerebral cortex after a few hours, which peaked approximately 24 h after injection. The later enrichment in subcortical white matter further support previous observations that CSF mediated tracer enrichment occurs from surface and inward (centripetal) (Ringstad et al., 2018). Significant clearance of the tracer may take up to 48 h or longer (Ringstad et al., 2023).

The present findings further showed that first glymphatic influx occurred somewhat earlier in IIH than REF subjects. Tracer enrichment in IIH was highest in the frontal, temporal, cingulate, insular, cerebellar and brainstem regions (Eide et al., 2021b). Moreover, the estimated first-time tracer appearance differed between REF and IIH for all regions but was significantly earlier in IIH for the temporal cerebral cortex and later for parietal and insular white matter and limbic structures (Table 2). These observations may align with earlier observations of more pronounced glymphatic influx in IIH patients, but using a different algorithm and statistical approach (Eide et al., 2021b). The IIH patients are characterized by increased pulsatile intracranial pressure (ICP) as well as abnormalities at the perivascular pathways of the brain (Eide, 2021; Eide and Hansson, 2022). Perhaps these abnormalities cause a facilitation of perivascular CSF-tracer influx in these cases. Our previous work on this cohort indicated most evident differences in frontal and temporal regions.

In the medical and health sciences, segmented regression is frequently applied in interrupted time series studies to assess trends before and after policy interventions (Schober and Vetter, 2021). It is typically used in quasi-experimental designs to evaluate health system quality improvement interventions when randomization is not possible or feasible (Bernal et al., 2017; Hategeka et al., 2020). While many interrupted time series studies use segmented regression analysis on time series data aggregated within periods, analysis of individual-level data is feasible using mixed effects models. Even though our data contained repeated measurements within each participant, we chose not to apply modeling using mixed-effects models to reduce complexity and due to the nature of our data. Especially, the somewhat low number of repeated measurements for each participant limited the use of this statistical method. We focused on the specific time of change in the T1 signal unit as a measure of the first appearance of the tracer after injection. Although it might be a statistical simplification, it ensures an efficient estimation of the breakpoint. However, the standard error and CI of the breakpoints might be affected by not considering repeated and clustered measurements within the same patient.

In previous studies, we have typically standardized relative to the pre-injection signal and grouped it into defined time intervals (Ringstad et al., 2018). These grouped data points were then evaluated using linear mixed model analysis for repeated measurements, with time as a categorical variable (Eide et al., 2021a). Due to variations in the MRI follow-up times across patients, this approach has proven to be statistically better for assessing differences over time and comparisons between patient groups at specified time intervals. Assessing the brain-wide CSF tracer enrichment profile requires the subject to be scanned at multiple time points. Additional scanning over time improves the precision of the CSF tracer profile. This can be readily performed in rodents but is not feasible in human studies (Boyd et al., 2024). For example, the exact time from injection to initial glymphatic enhancement, as detected by a change in the contrast signal, becomes less accurate when the time from injection is categorized into broader time intervals. Thus, grouping a continuous variable such as the observation time into predefined intervals can result in a loss of statistical information.

Repeated time-series analysis of glymphatic enrichment should therefore better estimate the period between the injection of the glymphatic tracer and its subsequent enhancement across different brain segments. Other advanced methods with robust change point detection for linear regression models, in the presence of potential outliers or with nonnormal structures (Alin et al., 2019), or recent advancements in linear mixed effects breakpoint analysis with robust estimation might improve estimation (Lee et al., 2024). However, we found that a simple statistical approach, combined with a thorough visual assessment of the data and a fitted regression model with an estimated breakpoint, was favorable in our study.

Although segmented regression with breakpoints seems to be a reasonable methodological approach, other statistical methods to assess nonlinear responses of glymphatic markers might be relevant. We used fractional polynomial regression analysis with robust standard errors for repeated measurements to explore daytime variations in plasma biomarker concentrations. This analysis was linked to the clearance of neurotoxic brain proteins via transfer from CSF to blood and the use of plasma biomarkers of neurodegeneration to predict neurological diseases (Eide et al., 2023a). However, in that study, a defined breakpoint was not expected; instead, a linear or nonlinear relationship with an unknown function was anticipated. Polynomial regression analysis was also applied to assess the mechanism behind changes in plasma neurodegeneration biomarkers induced by sleep deprivation (Eide et al., 2023b). The time from the intrathecal tracer injection to the first detection of the tracer around arteries and nearby cerebral cortex was visually assessed via MRI scans of the human PVSAS subarachnoid space without statistical modeling (Eide and Ringstad, 2024). However, to the best of our knowledge, there is no validated “gold standard” method for estimating the time of first tracer detection. Potentially, statistical modeling should improve the accuracy and precision of any estimates, including the time to first appearance of a glymphatic tracer.

### 4.1 Limitations

We acknowledge some limitations with the study. First, negative estimates (i.e., first-time detection before injection of the tracer) in the entorhinal cerebral cortex and choroid plexus in IIH patients could reflect methodological limitations or lower precision due to the small sample size. In both regions, the tracer appeared instantaneously after injection, and including two breakpoints or increasing the sample size might improve modeling in such cases. Second, it might be considered a limitation that the method cannot be used on the individual level. On the other hand, it may be used to compare patient cohorts to provide information at group level on possible disease mechanisms.

## 5 Conclusion

Segmented regression analysis seems to be a feasible method for estimating the time to first enrichment of a CSF tracer in brain regions after its intrathecal injection. This estimated time can serve as an indicator of glymphatic influx. Variations in methodological considerations and reporting require further investigation.

## Data Availability

The raw data supporting the conclusions of this article will be made available by the authors, without undue reservation.

## Footnotes

1
https://surfer.nmr.mgh.harvard.edu
