# Supplementary material for: A method for measuring first glymphatic influx of a cerebrospinal fluid tracer in the human brain
Source: Front Neurosci. 2025 Dec 19;19:1703748. doi: 10.3389/fnins.2025.1703748 (PMC12757395; doi:10.3389/fnins.2025.1703748)
Supplement: Supplementary file 1 [file Data_Sheet_1.pdf]

Supplementary material

A method for measuring first glymphatic influx of a cerebrospinal fluid tracer in the human brain

Are Hugo Pripp[1,2,3], Geir Ringstad[2,4,5,6], Lars Magnus Valnes[7,8] and Per Kristian Eide[2,6,7*]

[1] Oslo Centre of Biostatistics and Epidemiology, Oslo University Hospital, Oslo, Norway
[2] KG Jebsen Centre for Brain Fluid Research, University of Oslo, Norway
[3] Faculty of Health Sciences, OsloMet – Oslo Metropolitan University, Oslo, Norway
[4] Department of Radiology, Oslo University Hospital-Rikshospitalet, Oslo, Norway
[5] Department of Geriatrics and Internal Medicine, Sorlandet Hospital, Arendal, Norway
[6] Institute of Clinical Medicine, Faculty of Medicine, University of Oslo, Oslo, Norway
[7] Department of Neurosurgery, Oslo University Hospital-Rikshospitalet, Oslo, Norway
[8] Department of Mathematics, University of Oslo, Oslo, Norway

***Correspondence:**
Per Kristian Eide, MD PhD
Department of Neurosurgery
Oslo University Hospital - Rikshospitalet
Pb 4953 Nydalen
N-0424 Oslo
Norway
Phone: +47 91649419
E-mail: p.k.eide@medisin.uio.no

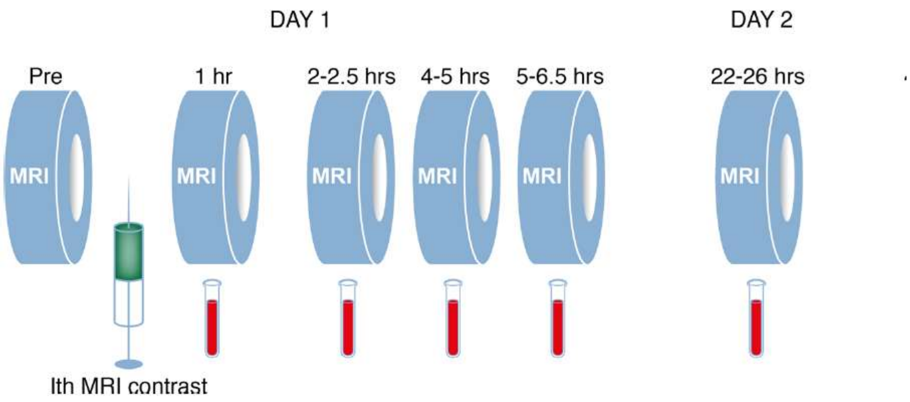

Supplementary Fig. 1 Study outline. A standardized protocol was followed for all study participants. T1-weighted MRI scanning was performed before the intrathecal (Ith) injection of the MRI contrast agent (Pre), and thereafter standardized T1-weighted MRI sequences were obtained at approximately 1, 2, 4, 6 and 24 hours. The illustration is adapted and reproduced from Eide PK, Mariussen E, Uggerud H, Pripp AH, Lashkarivand A, Hassel B, et al. Clinical application of intrathecal gadobutrol for assessment of cerebrospinal fluid tracer clearance to blood. JCI Insight. 2021;6(9). https://doi.org/10.1172/jci.insight.147063. (Published under the terms of the Creative Commons Attribution 4.0 International License, which allows for reuse without permission if the original source is cited).

Additional file 1. R code to estimate breakpoints from simulated data. These R code examples can be adapted for use in other studies. Furthermore, we present a basic assessment of true versus estimated breakpoints across three scenarios using simulated data.

R code to estimate the breakpoint in one group

```
library(segmented)

### Simulate data with one breakpoint ###
set.seed(123)    # Set seed for reproducibility
breakpoint <- 2  # Define breakpoint
time <- (seq(-2, 7, by=0.1))  # Generate time variable
signal <- ifelse(time <= breakpoint,
                 0.2 + 0 * time + rnorm(length(time),
                                        mean = 0, sd = 0.1),
                 0.2 + 0.1 * (time-breakpoint) + rnorm(length(time),
                                                       mean = 0, sd = 0.1))
df <- data.frame(time = time, signal = signal)

### Segmented regression modeling ###
lm <- lm(signal ~ 1,  data=df)
os<- segmented(lm, seg.Z=~time, npsi = 1)
summary(os)

### Time series plot ###
plot(df$time, df$signal, col="black", pch=1,
     xlab="Time (hours)",
     ylab="Signal")
plot(os, add=TRUE,  col="red",  lwd=5, cex=0.5, )
lines(os, col="red", bottom=FALSE)
```

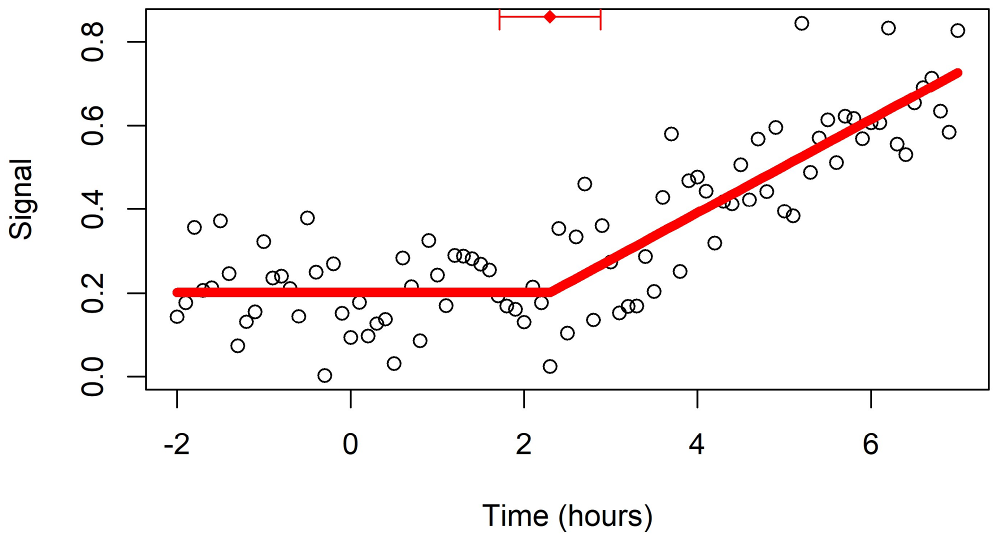

Supplementary Fig. 2 Time series plot with a segmented regression model to illustrate the estimation of first-time tracer appearance. The data are simulated

R code to estimate separate breakpoints in two groups

```
library(segmented)

### Simulate data with separate breakpoint of each group ###
set.seed(123)  # Set seed for reproducibility
breakpoint1 <- 1.5  # Define breakpoint of group 1
breakpoint2 <- 3    # Define breakpoint of group 2
time <- (seq(-2, 7, by=0.1))  # Generate time variable
signal <- ifelse(time <= breakpoint1,
                 0.2 + 0 * time + rnorm(length(time),
                 mean = 0, sd = 0.1),
                 0.2 + 0.1 * (time-breakpoint1) + rnorm(length(time),
                 mean = 0, sd = 0.1))
df1 <- data.frame(time = time, signal = signal, Group = 1)
signal <- ifelse(time <= breakpoint2,
                 0.2 + 0 * time + rnorm(length(time),
                 mean = 0, sd = 0.1),
                 0.2 + 0.1 * (time-breakpoint2) + rnorm(length(time),
                 mean = 0, sd = 0.1))
df2 <- data.frame(time = time, signal = signal, Group = 2)
df <- rbind(df1, df2)
df$Group <- factor(df$Group, levels=c(1,2),
                   labels=c("-Group1", "-Group2"))

### Segmented regression modeling ###
os<-segreg(signal~ 1 + seg(time, npsi=1,
                           by=Group, est=c(0,1)), data=df)
summary(os)

### Time series plot ###
plot(df$time, df$signal, col=1:2, pch=1,
     xlab="Time (hours)",
     ylab="Signal")
plot(os, add=TRUE,  term=1:2, col=1:2,
     lwd=5, cex=0.5, leg="left")
lines(os, term=1 ,col=1, bottom=FALSE, k=50)
lines(os, term=2 ,col=2, bottom=FALSE, k=20)
```

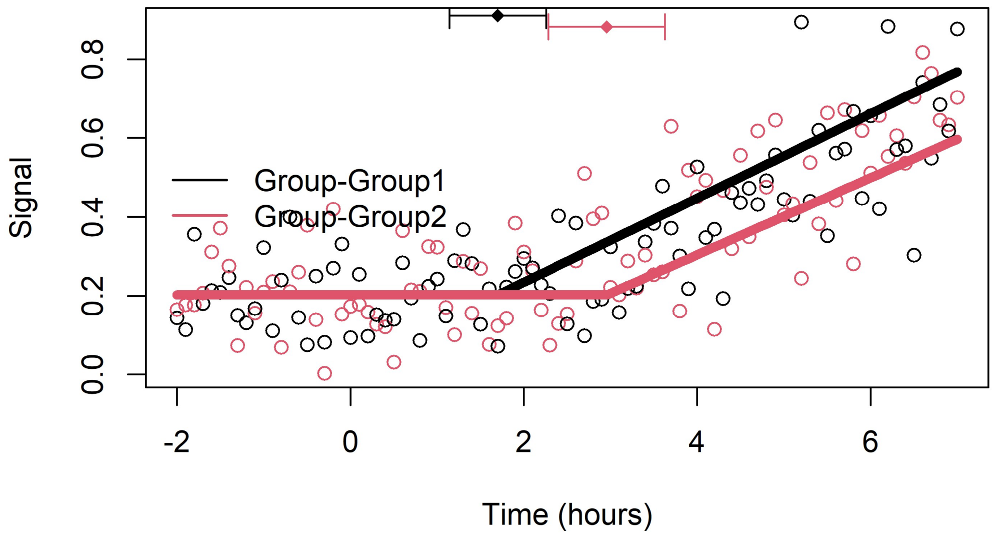

Supplementary Fig. 3 Time series plot with a segmented regression model to estimate the first-time tracer appearance in two separate groups with a common horizontal line before tracer injection. The data are simulated.

True versus estimated breakpoints with an increasing number of data points (n) and different time distributions.

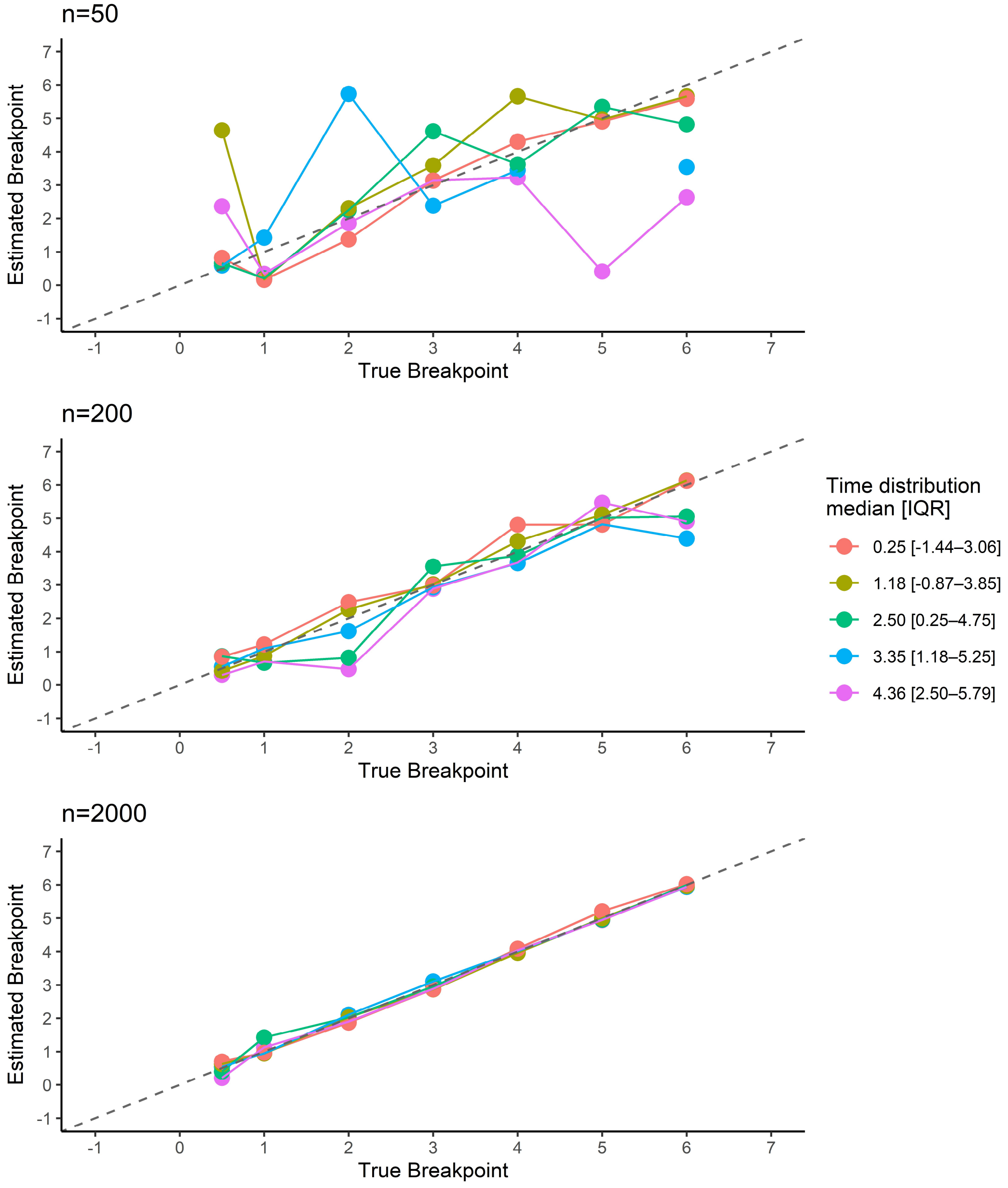

Supplementary Fig. 4 Using the model in Supplementary Fig. 1, we show the relationship between true and estimated breakpoints in scenarios with 50, 200, and 2,000 data points and different time distributions. A median time distribution with interquartile range (IQR) presented as the 1st and 3rd quartiles of 2.50 [0.25–4.75] represents a uniform distribution (i.e., equally spaced) from 2 hours before to 7 hours after injection.
